# Long-standing temporomandibular joint dislocation treated by intraoral condylectomy: a case report and review of the literature

**DOI:** 10.1186/s13256-022-03471-y

**Published:** 2022-06-23

**Authors:** Ryo Uetsuki, Shigehiro Ono, Misato Tada, Satoshi Okuda, Masaaki Takechi

**Affiliations:** Department of Oral and Maxillofacial Surgery, Graduate School of Biomedical & Health Sciences, Hiroshima University, Kasumi 1-2-3, Minami-ku, Hiroshima, 734-8553 Japan

**Keywords:** Long-standing TMJ dislocation, Intraoral condylectomy, Noninvasive reduction

## Abstract

**Background:**

Noninvasive management by closed reduction is a desirable treatment for temporomandibular joint dislocation. However, reduction of long-standing temporomandibular joint dislocation is often difficult. Various conservative treatments have been attempted, but these often render poor outcomes. This article reports the case of long-standing temporomandibular joint dislocation that was successfully closed using intraoral condylectomy.

**Case presentation:**

A 69-year-old Japanese man who sustained an injury in a car collision was unable to close his mouth. Owing to the diagnosis of long-standing temporomandibular joint dislocation, intraoral condylectomy was performed. In the case of temporomandibular joint dislocation, it is convenient to reach the condyle from the oral cavity because sufficient opening is maintained. The condyle can be clearly visualized using an approach similar to sagittal split ramus osteotomy, and the operation using surgical instruments can be facilitated by resecting the coronoid process. By separating the surrounding soft tissue and pulling the cut condyle with sufficient visual field, the condyle can be resected while addressing the hemostasis. During the 12-month postoperative follow-up period, no temporomandibular joint dislocation recurred and the occlusion remained stable.

**Conclusions:**

The limited intraoral incision of this surgical technique provides sufficient access for condylectomy. The results of this case report suggest that condylectomy by intraoral approach could become the treatment of choice for long-standing temporomandibular joint dislocation.

## Background

Dislocation of the temporomandibular joint (TMJ) results in a locked position of the condyle anterior to the tuberculum articulare [1]. This may occur either unilaterally or bilaterally. Common causes of TMJ dislocation include intubation procedure, gastrointestinal endoscopies, and trauma. Long-term dislocation of the TMJ is defined as an acute dislocation left untreated or inadequately treated for more than 72 hours [2]. The prolonged inability to close the mouth results in difficultly in eating and speaking, xerostomia, and significant reduction of quality of life. Noninvasive treatment is effective for TMJ dislocation; however, the reduction of long-standing TMJ dislocation is often difficult. We report a case of long-standing TMJ dislocation that was successfully closed using intraoral condylectomy.

## Case presentation

**Fig. 1:**
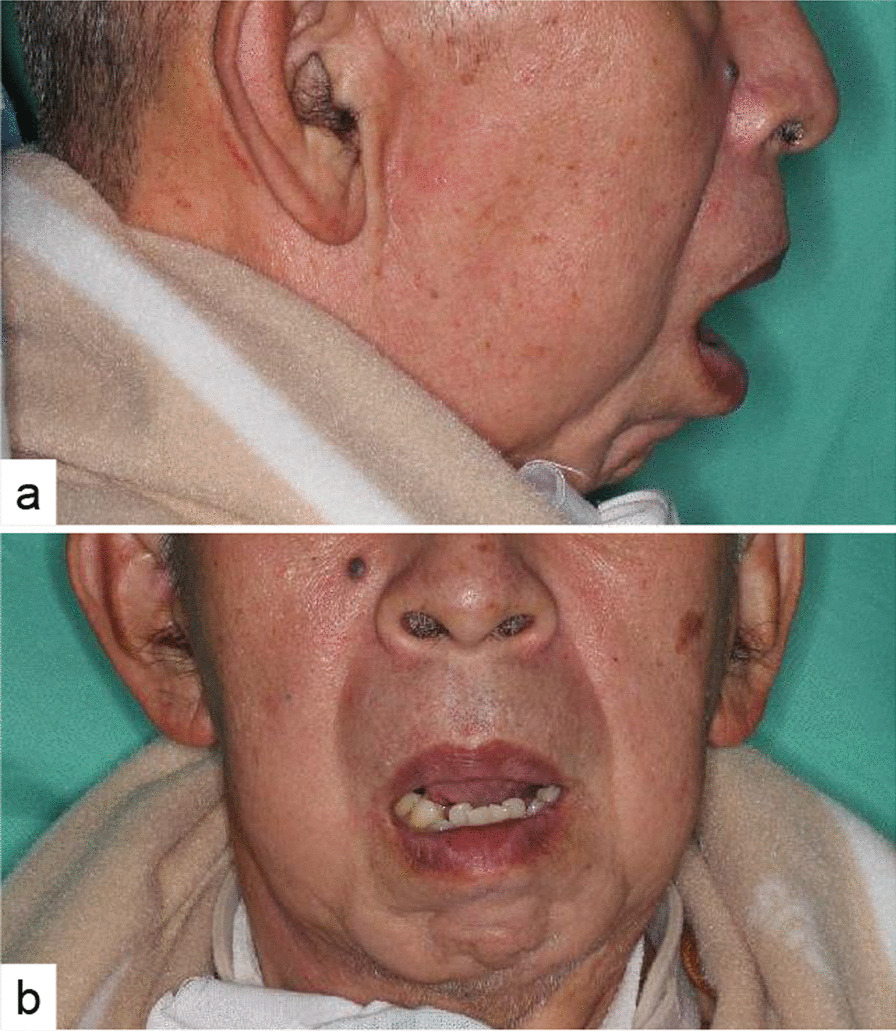
Facial photographs before treatment. The jaw cannot be closed, and the chin is located in front. **a** Lateral view; **b** frontal view

A 69-year-old Japanese man who sustained an injury in a car collision received first aid at a nearby hospital and underwent craniotomy surgery for traumatic acute subdural hematoma, traumatic subarachnoid hemorrhage, and traumatic localized brain contusion. Conservative treatment was given for left-sided condylar process fracture, axis fracture, right clavicle fracture, and right fracture of the distal radius. After his consciousness disorder improved, 4 months after injury, it became clear that the patient was unable to close his mouth. His TMJ may have been dislocated during intubation. The mouth presented in an open state, with lip closure incapacity (Fig. 1a, b). Stenosis of the jaw dentition was demonstrated with labioclination of the front tooth, open bite, and xerostomia. The patient had a tracheostomy and could not evaluate the verbal response, Glasgow Coma Scale (GCS) 11. Traumatic brain injury left paralysis of his right upper extremity. The patient was at high risk of aspiration pneumonia due to diminished swallowing function, so the previous doctor had performed an additional gastrostomy, which recovered his ability to eat.

**Fig. 2:**
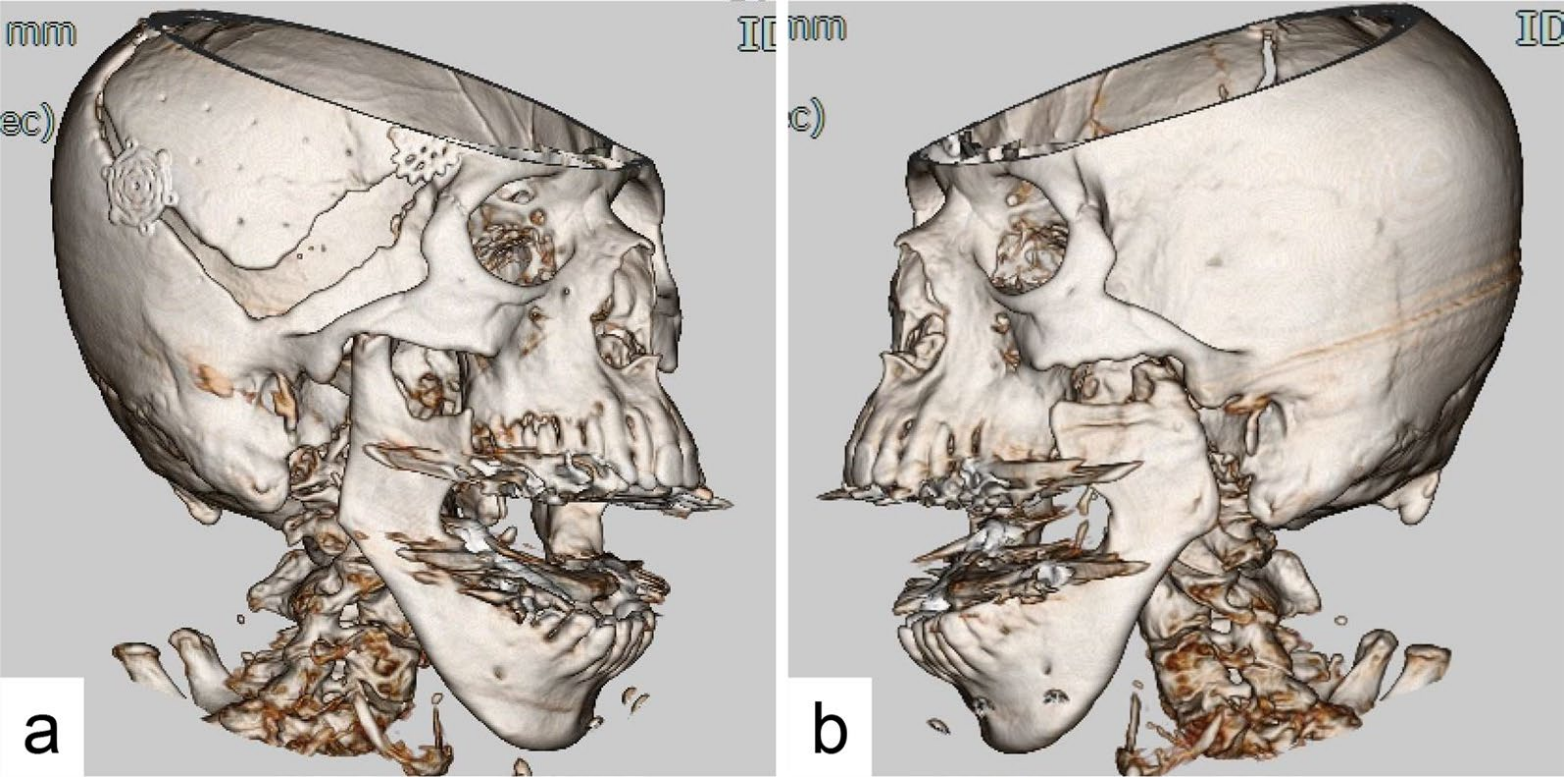
Computed tomography at pretreatment. The left condylar process fracture on the medial side. Bilateral mandibular condyles greatly exceeded the articular tubercle. After craniotomy operation of the temporal region at right side. **a** Right side; **b** left side

Panoramic radiography examination revealed a left condylar process fracture and dislocation of the bilateral mandibular condyles. On computed tomography examination, the left condylar process fracture on the medial side and both mandibular condyles greatly exceeded the articular tubercle (Fig. 2a, b).

We attempted a manual reduction owing to the existing diagnosis of old left condylar fracture and bilateral anterior dislocation of the TMJ, but reduction was impossible. Therefore, we planned to perform right condylar resection using an intraoral approach. In addition, we evaluated the planned procedure using a three-dimensional model and confirmed that it would not disturb the jaw reposition, because the left condylar process fractures to medial. We confirmed that it was difficult to reduce and fix the old fracture and that it did not interfere with the reproduction of the original occlusion on the three-dimensional model, so we decided to treat it conservatively.

**Fig. 3:**
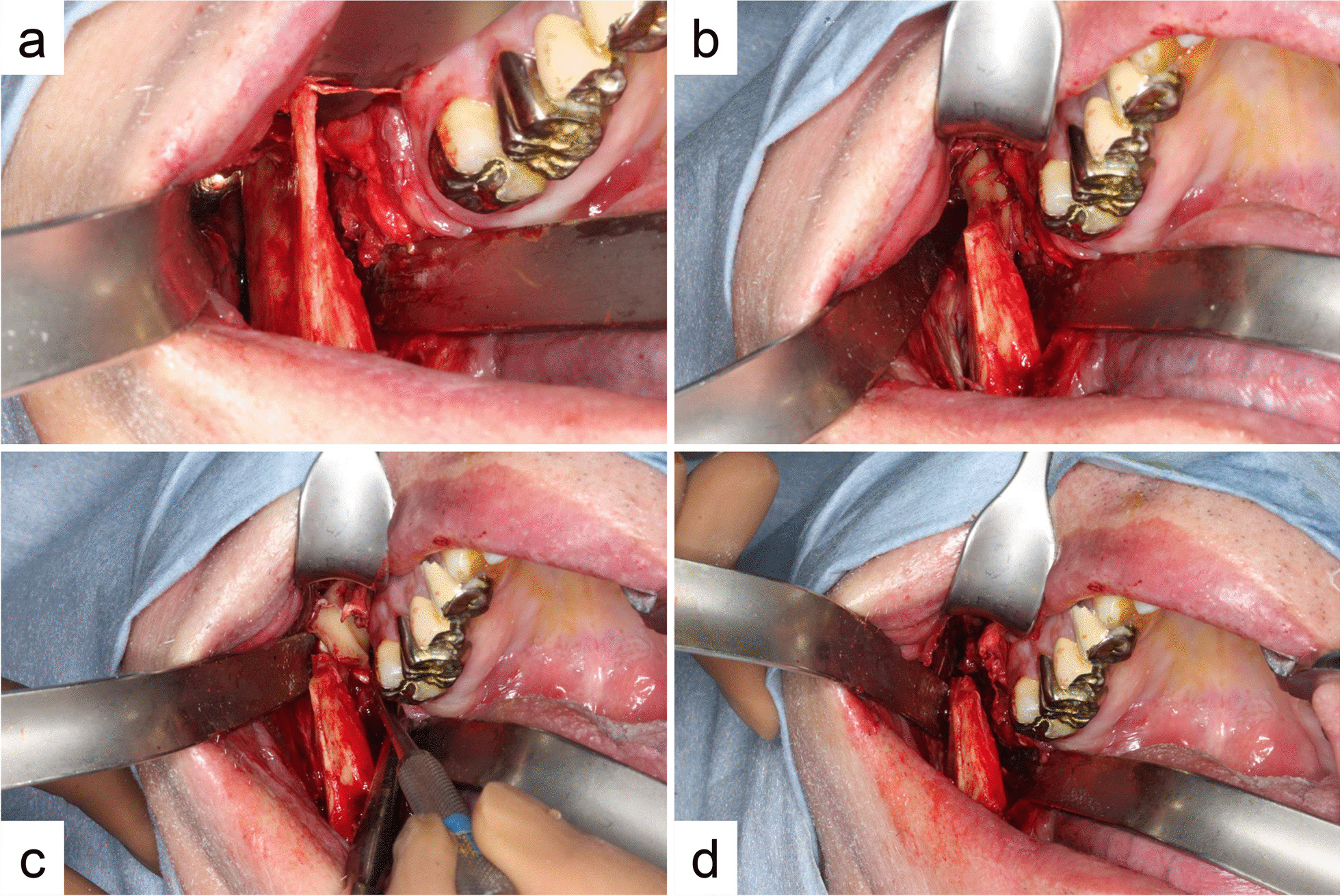
Right coronoid process and the neck of the mandible (**a**). After coronoid process resection (**b**). The base of the condylar was cut using a saw and osteotome (**c**). After condylectomy (**d**)

**Fig. 4:**
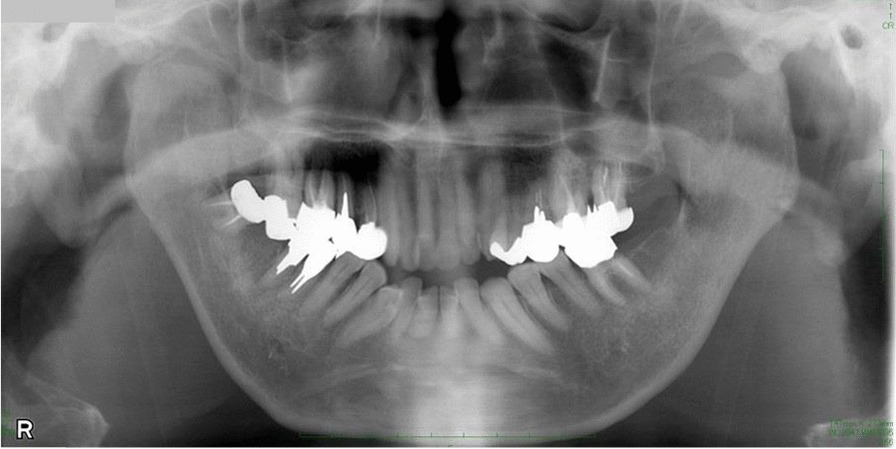
Postoperative panoramic radiographs of the patient. No temporomandibular joint dislocation recurrence

We performed right condylectomy with the patient under general anesthesia. Manual reduction was performed again in a state of muscle relaxation, but reduction was not possible. After an incision was made in the buccal mucosa according to the sagittal split ramus osteotomy, the inside and outside of the mandibular ramus was revealed, and the coronoid process and the neck of the mandible were confirmed (Fig. 3a). The coronoid process obstructed the visual field, so it was cut and resected with a reciprocating saw (Fig. 3b). Using a saw and osteotome, the base of the condylar was cut and separated from the surrounding soft tissue to remove the condylar head (Fig. 3c, d). We confirmed that the mandibular body had moved backward and that the molars could achieve the occlusion, and the wound was closed. Intermaxillary traction was started during the postoperative period using an intermaxillary fixation screw inserted in the alveolar region to improve occlusal deviation. Twenty-one days after the operation, a normal occlusion was obtained, and intermaxillary fixation screw was removed. During the 12-month postoperative follow-up period, no TMJ dislocation recurred, and the occlusion remained stable (Fig. 4). After the operation, he was able to open and close the mouth by himself, and the lips could be closed, so that hypersalivation did not occur. His left condylar process was displaced inward owing to a fracture, but the opening and closing movements were possible because the hinge movement centered on the left condylar process.

## Discussion

TMJ dislocation is a condition in which the mandibular condyle deviates from the mandibular fossa and cannot be reduced on its own [1]. Symptoms, such as inability to close the mouth, hypersalivation and mastication disorder, and dysarthria, cause remarkable deterioration in the patient’s quality of life. For this reason, we often treat these cases early; however, treatment can become obsolete when the consciousness is impaired, as it was in this case. In long-standing TMJ dislocation, manual reduction is extremely difficult because the mandibular fossa is filled with connective tissue-like structures and scar-like tissue [3]. In this case, it was difficult to perform manual reduction, and we expected that the connective tissue-like structure was filled in the mandibular fossa.

Noninvasive reduction is desirable for the treatment of long-standing TMJ dislocation. Conservative treatment of a previous dislocation of the TMJ includes manual reduction and reduction using a lever action [4]. It has been reported that the lever action slowly relieves the soft tissue that fills the mandibular fossa, reduces the condyle, and prevents re-dislocation by tissue repair. In this case, we considered reduction by lever action. However, because we were concerned that deterioration in oral hygiene caused by wearing the treatment device for several weeks could cause aspiration pneumonia, we chose open surgery.

There have been many reports of the use of both arthroscopic surgery [5] and Fink’s method [6] for open reduction of a previous dislocation of long-standing TMJ dislocation. Fink’s method reduces dislocation by hooking the sigmoid notch with a single hook and pulling it downward. If reduction is not possible using these treatments, resection of the soft tissue in the mandibular fossa or condylectomy to reduce the interference with soft tissue in the mandibular fossa is indicated. In this case, dislocation of the TMJ continued over a long period of time, and manual reduction was difficult. Thus, we expected that soft tissue was present in the mandibular fossa. Therefore, resection of the soft tissue in the mandibular fossa [7, 8] or condylectomy [9, 10] was considered an indication for open surgery.

An extraoral approach is commonly used in cases in which soft tissue in the mandibular fossa is resected or condylectomy is performed while the joint cavity is open. An incision from the preauricular to the temporal region, as represented by the Al-Kayat–Bramley method [11], has been reported to secure a wide surgical field and prevent nerve and vessel injuries. However, in this case, we avoided making an incision from the preauricular to the temporal region because the surgical field overlapped with that of the craniotomy operation of the temporal region. Condylectomy has been reported to be performed using an intraoral method [12, 13] in addition to an extraoral approach. The use of intraoral condylectomy can avoid the complications of the extraoral approach. However, there is a risk of damage to the maxillary artery, pterygoid plexus, and retromandibular vein. Vascular damage can be avoided by opening a large opening and performing surgery under direct vision. In addition, we need to be careful about postoperative infections. The loss of temporalis function due to coronoid process dissection is expected to be supplemented by the masseter and medial pterygoid muscles. The advantages of intraoral condylectomy are the absence of a facial scar and the reduced possibility of nerve and vessel injury.

## Conclusion

In the case of TMJ dislocation, it is easier to reach the condyle from the oral cavity because a sufficient opening is maintained. The condyle can be clearly visualized using the same approach as in sagittal split ramus osteotomy, and the operation using surgical instruments can be facilitated by resecting the coronoid process. By separating the surrounding soft tissue and pulling the cut condyle with sufficient visual field, the condyle can be resected while responding to bleeding. This intraoral approach could become the treatment of choice for long-standing TMJ dislocation.

## Data Availability

Not applicable

## Abbreviation

TMJ: Temporomandibular joint
